# Non-functional alpha-cell hyperplasia with glucagon-producing NET: a case report

**DOI:** 10.3389/fendo.2024.1405835

**Published:** 2024-09-06

**Authors:** Catarina Cidade-Rodrigues, Ana Paula Santos, Raquel Calheiros, Sara Santos, Catarina Matos, Ana Paula Moreira, Isabel Inácio, Pedro Souteiro, Joana Oliveira, Manuel Jácome, Sofia S. Pereira, Rui Henrique, Isabel Torres, Mariana P. Monteiro

**Affiliations:** ^1^ Hospital Padre Américo, Unidade Local de Saúde do Tâmega e Sousa, Penafiel, Portugal; ^2^ Department of Endocrinology, Portuguese Oncology Institute of Porto (IPO Porto), Porto, Portugal; ^3^ Research Center of IPO Porto (CI-IPOP), RISE@CI-IPO (Health Research Network), Portuguese Oncology Institute of Porto (IPO Porto), Porto Comprehensive Cancer Centre (P.CCC), Porto, Portugal; ^4^ Hospital de Braga, Unidade Local de Saúde de Braga, Braga, Portugal; ^5^ Institute for Nuclear Sciences Applied to Health (ICNAS), University of Coimbra, Coimbra, Portugal; ^6^ Department of Pathology, Portuguese Oncology Institute of Porto (IPO Porto), Porto, Portugal; ^7^ Unit for Multidisciplinary Research in Biomedicine (UMIB), School of Medicine and Biomedical Sciences (ICBAS), University of Porto, Porto, Portugal; ^8^ Laboratory of Integrative and Translocation Research in Population Health (ITR), Porto, Portugal; ^9^ Department of Pathology and Molecular Immunology, School of Medicine and Biomedical Sciences (ICBAS), University of Porto, Porto, Portugal

**Keywords:** alpha-cell, hyperplasia, neuroendocrine tumors, glucagon-producing NET, pancreas

## Abstract

**Introduction:**

Alpha-cell hyperplasia (ACH) is a rare pancreatic endocrine condition. Three types of ACH have been described: functional or nonglucagonoma hyperglucagonemic glucagonoma syndrome, reactive or secondary to defective glucagon signaling, and non-functional. Few cases of ACH with concomitant pancreatic neuroendocrine tumors (pNETs) have been reported and its etiology remains poorly understood. A case report of non-functional ACH with glucagon-producing NET is herein presented.

**Case report:**

A 72-year-old male was referred to our institution for a 2 cm single pNET incidentally found during imaging for acute cholecystitis. The patient’s past medical history included type 2 diabetes (T2D) diagnosed 12 years earlier, for which he was prescribed metformin, dapagliflozin, and semaglutide. The pNET was clinically and biochemically non-functioning, apart from mildly elevated glucagon 217 pg/ml (<209), and ^68^Ga-SSTR PET/CT positive uptake was only found at the pancreatic tail (SUVmax 11.45). The patient underwent a caudal pancreatectomy and the post-operative ^68^Ga-SSTR PET/CT was negative. A multifocal well-differentiated NET G1, pT1N0M0R0 (mf) strongly staining for glucagon on a background neuroendocrine alpha-cell hyperplasia with some degree of acinar fibrosis was identified on pathology analysis.

**Discussion and conclusion:**

This case reports the incidental finding of a clinically non-functioning pNET in a patient with T2D and elevated glucagon levels, unexpectedly diagnosed as glucagon-producing NET and ACH. A high level of suspicion was required to conduct the glucagon immunostaining, which is not part of the pathology routine for a clinically non-functioning pNET, and was key for the diagnosis that otherwise would have been missed. This case highlights the need to consider the diagnosis of glucagon-producing pNET on an ACH background even in the absence of glucagonoma syndrome.

## 1 Introduction

Pancreatic endocrine cell hyperplasia may occur in approximately 0.6% of adults (1), while isolated alpha-cell hyperplasia (ACH), a condition that was first reported in 1991, is even more rare (2). ACH etiology remains poorly understood (2), but has been described to affect both women and men with an age at diagnosis ranging from 25 to 74 years old (3).

Three types of ACH have thus far been described: functional, reactive, and non-functional (3).

Functional ACH represents 17% of cases (3) and presents with nonglucagonoma hyperglucagonemic glucagonoma syndrome. Therefore, glucagon levels are elevated, but no gross pancreatic neuroendocrine tumor (pNET) is found (4, 5).

Reactive ACH is the most frequent type, accounting for 42% of cases and results from defective glucagon receptor signaling, either due to inactivating mutations or deletions of the glucagon receptor gene or glucagon receptor intracellular pathways, also known as Mahvash disease. It may present with nonspecific symptoms including weight loss, abdominal pain or altered intestinal transit, or as an incidentally discovered pancreatic mass. This ACH subtype is characterized by marked hyperglucagonemia without glucagonoma syndrome, as well as the development of gross pNETs (3).

Non-functional ACH is responsible for 25% of cases (3) and is characterized by normal or slightly elevated glucagon levels. It may also present with nonspecific symptoms, although glucagonoma syndrome is usually absent. ACH is most often an incidental histological finding and its clinical relevance is the putative increased risk for pNETs.

Only a few cases of ACH associated with pNETs have been described in the literature. Among these, some reported the association of reactive ACH with glucagonoma (4, 6, 7) or a non-functioning pNET (8).

A case report of the incidental diagnosis of a non-functional ACH with a glucagon-producing NET is herein presented.

## 2 Case description

We present a 72-year-old male individual, referred to our tertiary reference center for a single 2 cm pNET incidentally discovered in a CT scan performed during an investigation for acute cholecystitis. The tumor was clinically non-functioning as symptoms or signs of hormone hypersecretion were absent.

The patient’s past medical history included type 2 diabetes (T2D) diagnosed 12 years earlier, arterial hypertension, and dyslipidemia. T2D glycemic control over the years was overall good, with HbA1c levels ranging between 5 and 6%, while prescribed metformin 1000 mg bid and dapagliflozin 10 mg od. Furthermore, he had received treatment with once-weekly semaglutide 1mg for 3 months, 1 year before surgery. The patient had no past medical history of pancreatitis. Family history was irrelevant.

The patient’s body mass index was 25.6 kg/m^2^, but physical examination was otherwise unremarkable, without palpable abdominal masses or skin lesions.

Biochemical analysis showed an HbA1c 6%, insulin 5.15 uUI/ml (2.6-24.9), somatostatin < 3.9 pmol/l (< 16.0), gastrin 12.8 pg/ml (< 108), vasoactive intestinal peptide 15.7 pmol/l (< 30) and mildly elevated glucagon [217 pg/ml (<209)], as presented in **Table 1**. Glucagon levels were measured using a radioimmunoassay (RB310, DIAsource ImmunoAssays, S.A., Belgium), which exhibited a cross-reactivity of less than 0.1% with glucagon-related peptides.

**Table 1:** Initial blood test evaluating tumor secretion status.

Serum Parameter	Result (reference range)
Glucose	86 mg/dL (< 100)
Creatinine	0.6 mg/dL (0.7-1.3)
Aspartate aminotransferase	14 U/L (8-33)
Alanine transaminase	15 U/L (7-56)
HbA1c	5.4% (< 5.7)
TSH	5.31 µUI/mL (0.4-5.5)
Chromogranin A	41 ng/mL (< 98.1)
**Glucagon**	217 pg/mL (< 209)
Insulin	5.15 uUI/mL (2.6-24.9)
C-peptide	2.45 ng/mL (1.1-5.0)
Somatostatin	< 3.9 pmol/L (< 16.0)
Gastrin	12.8 pg/mL (< 108)
VIP	15.7 pmol/L (< 30)
ACTH	38.5 pg/mL (7.2-63.3)
Ionized calcium	1.26 mmol/L (1.17-1.38)
IGF-1	66.2 ng/mL (61-186)
PTH	28.9 pg/mL (12-65)
Prolactin	7.36 ng/mL (< 20)

**Figure 1:**
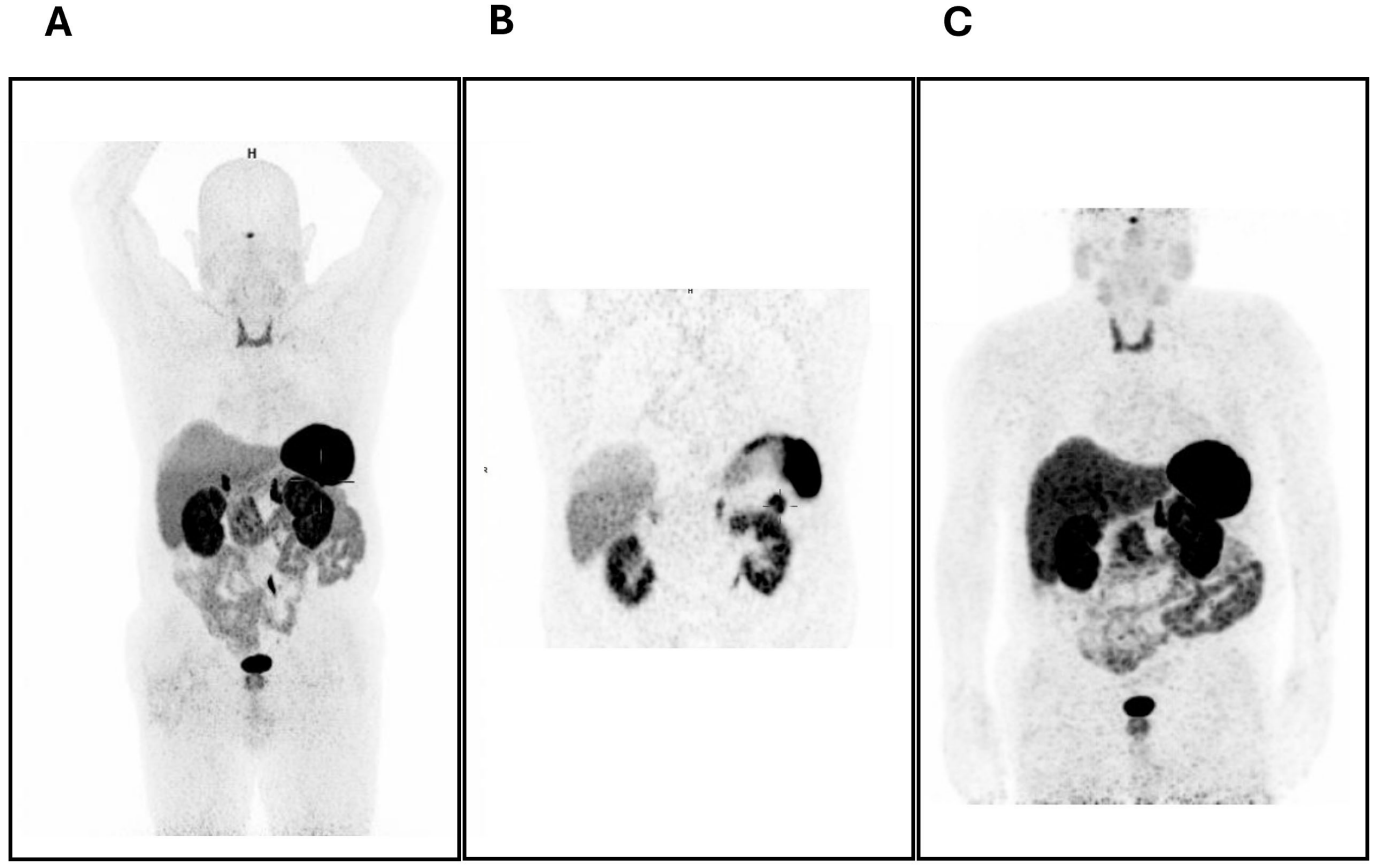
**(A, B)** 68Ga-DOTA-NOC PET/CT scan before treatment showing the positive uptake in the pancreatic tail (SUV max 11.45). **(C)** 68Ga-DOTA-NOC PET/CT scan after surgery showing no hyperfixation foci.

Abdominal magnetic resonance imaging (MRI) showed a nodule in the tail of the pancreas measuring approximately 25mm in its longest axis. It was difficult to delineate, showing a hyposignal on T2-weighted images and isosignal on T1-weighted images, with increased enhancement in relation to the surrounding pancreatic parenchyma. This was in favor of the hypothesis of a neuroendocrine tumor. ^68^Ga-SSTR PET*/*CT showed positive uptake in the pancreatic tail (SUV max 11.45), as shown in **Figure 1**. The patient underwent a caudal pancreatectomy with curative intent, which occurred without complications, and post-operative CT and ^68^Ga-SSTR PET*/*CT were negative, as shown in **Figure 1**.

**Figure 2:**
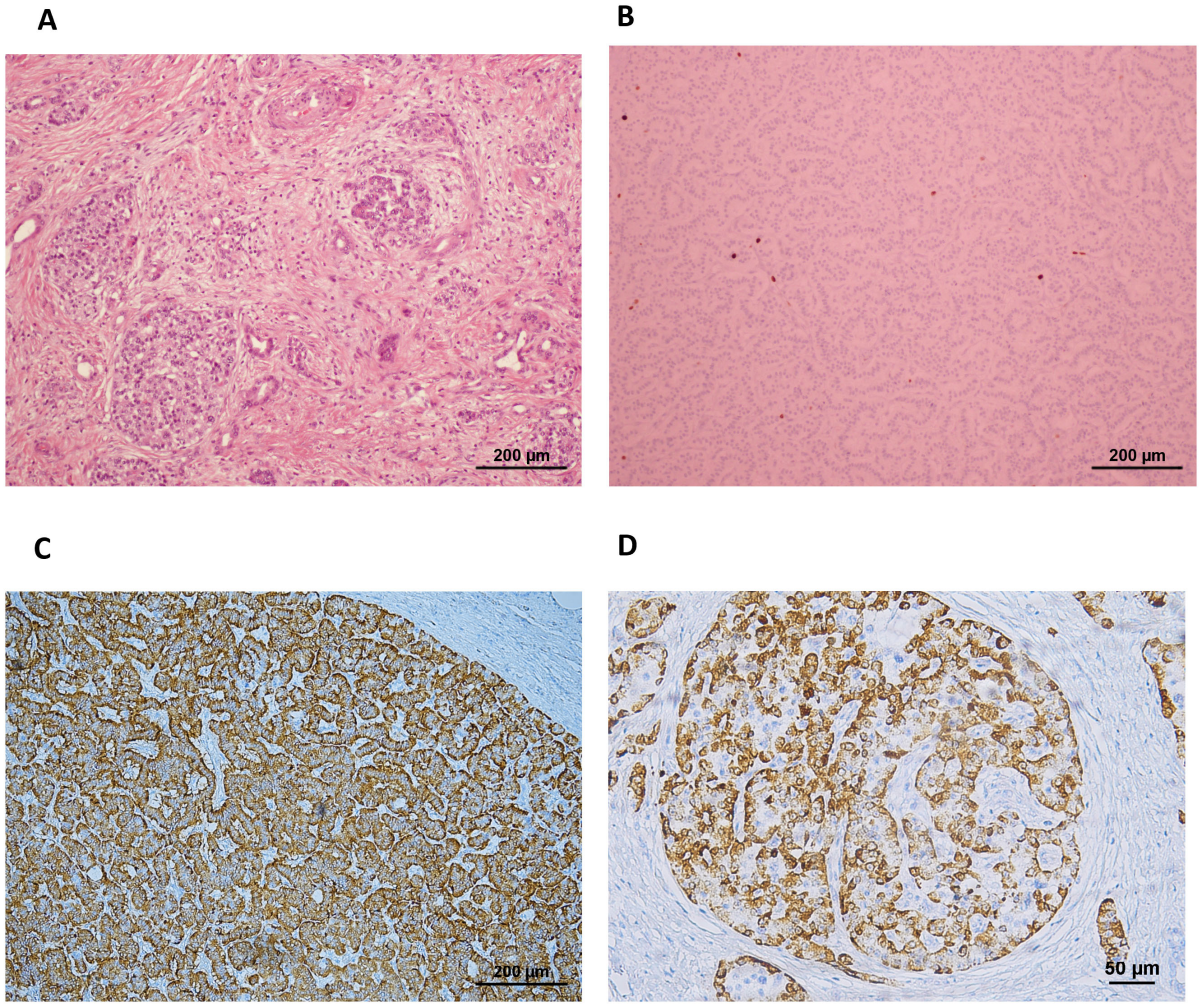
**(A)** H&E staining showing the pancreas with multiple scattered foci of fibrosis and **(B)** neuroendocrine cell hyperplasia including a pNET with low Ki67 labeling. **(C)** The pNET was strongly positive for staining for glucagon (ref. 565860, BD Pharmingen) on immunohistochemistry, and **(D)** the pancreatic islets depicted over 80% of the cells staining positive for glucagon, while the percentage in normal human islets is usually under 50% even considering the larger relative proportion observed in pancreas from individuals with T2D (9).

On macroscopical examination, an area with a nodular outline was identified, with a slightly increased consistency, a yellowish-white color, and imprecise boundaries. The 25mm nodule disclosed on MRI largely corresponded to chronic pancreatitis lesions. The largest tumor focus identified microscopically had 0.4cm in at the greatest diameter and corresponded to a multifocal well-differentiated neuroendocrine tumor G1 pT1N0M0R0 (mf), strongly staining for glucagon. The pancreas presented multiple and scattered foci of fibrosis and atrophy of the exocrine pancreas, associated with neuroendocrine hyperplasia. Mitotic figures were scarce (<1 per 2mm^2^) and Ki67 labeling was less than 3%. Lymphovascular or perineural invasion was not found. On immunohistochemistry, neoplastic cells disclosed immunoreactivity for cytokeratin 8/18, chromogranin, synaptophysin, and glucagon. Pancreatic islets presented a glucagon-producing α-cells percentage superior to 80%, while the percentage in normal human islets is usually under 50% even considering the larger relative proportion observed in the pancreas of individuals with T2D (9). These findings were compatible with the pathological diagnosis of glucagon-producing NET and ACH (**Figure 2**).

No specific genetic analysis for hereditary cancer syndromes was performed given the negative family history.

The patient remains recurrence- and symptom-free at 9 months of follow-up after surgery.

## 3 Discussion

This case reports the incidental finding of a glucagon-producing NET in the absence of the typical glucagonoma syndrome on a background of ACH.

ACH has been thought to be a potential preneoplastic condition, in particular the reactive and non-functional subtypes, while it remains to be proven whether the same applies to the functional subtype. Furthermore, ACH is characterized by a diffuse and specific increase in alpha-cell mass on histological analysis, which differs from the nonspecific alpha- and beta-cell hyperplasia found in patients with multiple endocrine neoplasia type 1 and von Hippel-Lindau syndrome (10–12).

The most well-characterized type is reactive ACH, since there is an identified molecular defect in the glucagon signaling pathway that can be replicated in an animal model (13, 14). In GCGR -/- mice, the progression from pancreatic endocrine cell mass expansion with ACH to dysplasia at 5 to 7 months, proceeding to pNETs < 1 mm at 12 months and then to pNETs > 1 mm at 18 months is well-established (13, 14). Moreover, the majority of these pancreatic lesions express glucagon, although some may also express insulin or no hormones (13, 14). In contrast, the pathogenesis of functional and nonfunctional ACH, for which there are no animal models that could speed the process of unraveling the mechanisms of disease, remains obscure. Nevertheless, besides reactive ACH and at least for nonfunctional ACH, the possible contribution of hyperplastic alpha-cells and their morphological changes for the development of pNETs remains a reasonable hypothesis.

There are no specific treatment recommendations for reactive or nonfunctional ACH currently established (13, 14). Therefore, for pNETs arising within the framework of reactive and nonfunctional ACH, treatment follows the same recommendations as for sporadic pNETs occurring outside the ACH context (15). Observational surveillance could be considered for nonfunctioning tumors ≤ 2 cm. Surgical resection is the preferred treatment for non-metastatic tumors larger than 2 cm, whether these are functioning or nonfunctioning. Subtotal or total pancreatectomy has been advocated as a treatment approach for functional ACH (3).

For the patient herein described, surveillance could have been an option given that the pNET was apparently non-functioning with a size of 25 mm. However, taking into account the patient’s preference and given the borderline tumor size and easy surgical access (caudal), the multidisciplinary team’s decision was in favor of distal pancreatectomy surgery. Otherwise, ACH would not have been detected nor would the glucagon-producing NET have been diagnosed.

Given the concurrent diagnosis of diabetes, two questions emerge: could diabetes be a manifestation of subclinical hyperglucagonemia? Alternatively, could ACH have been elicited by the use of incretin-based anti-diabetes medications, namely *g*lucagon*-*like peptide-1 receptor agonists (GLP-1Ra), subsequently contributing to the development of glucagon-producing NET?

Regarding the first question, diabetes possibly being secondary to glucagon excess is a reasonable hypothesis since glucagon seems to be more critical for the development or worsening of diabetes than insulin deficiency (16–18). Glucagon is able to induce liver glycogenolysis and raise blood glucose levels through specific binding to its receptor (GCGR) (18, 19). Indeed, the mild and subclinical hyperglucagonemia to which the patient was exposed could provide an explanation for the good glycemic control observed over more than a decade. Typical glucagonoma syndrome includes diabetes, necrolytic migratory erythema, depression, and deep vein thrombosis in the presence of hypoaminoacidemia and high glucagon levels, usually > 1000pg/ml (20–22). In fact, glucagon-producing NETs are usually larger than 4 or 5 cm in diameter at diagnosis and, at that stage, are often associated with locoregional or even distant metastases at diagnosis (20, 21). This notwithstanding, some patients recall the presence of a few selected nonspecific features for many years before clinical deterioration coincident with the development of the classic glucagonoma constellation (23). In the herein reported case, since there was only a mild hyperglucagonemia without typical glucagonoma syndrome features, a non-functional pNET with unrelated concomitant hyperglucagonemia cannot be excluded. Although the cause for the hyperglucagonemia remains otherwise undisclosed given that the patient’s past medical history was unremarkable for pancreatitis, liver, or renal impairment.

Regarding the second question, whether GLP-1Ra could have elicited ACH, this is even less likely. Indeed, GLP-1Ra are recognized to reduce glucagon levels by 20% to 50% through a strong and conserved negative feedback mechanism and consequently a reactive alpha-cell hyperplasia occurs (24). However, there is no evidence of any association between this histological finding and the risk of pNET development. Additionally, although *in vitro* and *in vivo* animal studies native GLP-1 was shown to promote pancreatic beta-cell proliferation, inhibition of its apoptosis, and differentiation of stem cells in the ductal epithelium through neogenesis in the islets (25), the same was not demonstrated to occur in humans, nor is it supported by the extensive data derived from clinical trials across the GLP-1Ra drug class (26, 27). Nonetheless, the putative long-term risk for exocrine and endocrine pancreatic neoplasia associated with incretin-based drugs has been a matter of concern, although highly controversial since the available evidence is scarce and mostly derived from animal studies or small human case series involving exenatide (25, 28, 29) and irrespective of the presence of ACH. Therefore, the hypothesis that GLP-1Ra might accelerate the progression of pancreatic dysplastic lesions, especially in the context of pre-existing chronic pancreatitis, a known predisposing factor for exocrine pancreatic cancer, and premalignant pancreatic intraepithelial neoplasia (28, 30), cannot be entirely excluded (31). However, there is no available evidence that GLP-1Ra could trigger glucagon-producing or other non-functioning pNETs.

In human *pancreata* obtained at autopsy, Butler A et al. found that individuals with T2D treated with incretin bases therapies (exenatide and sitagliptin) exhibited a 40% increase in pancreatic mass when compared to those with T2D not treated with these drugs. The authors also found higher rates of exocrine and endocrine cell proliferation and a higher prevalence of pancreatic intraepithelial neoplasia and ACH. Furthermore, among the eight individuals studied, three presented with adenomas smaller than 1 cm and 1 had an adenoma ≥ 1 cm, the majority of which stained positive for glucagon (29). It is important to note that this study was a small case series, which included only one patient treated with the GLP-1Ra exenatide (29).

Incretin-based therapies are also a cause of concern for increased risk of exocrine pancreatic neoplasms. In patients treated with GLP-1Ra, acute and chronic pancreatitis, whether clinical or subclinical, seems to ensue from duct cell proliferation and obstruction, as a predisposing factor to exocrine pancreatic cancer (25). As such and given the fact that incretin-based therapies were first introduced for T2D treatment in 2005, there is still no data regarding the true impact of these drugs in the risk for these types of tumors, since the effect present is probably too small to be detected. It should be stressed that up to this date, only exenatide has been associated with an increased risk of pancreatic cancer. Moreover, a large number of randomized clinical trials, as well as real-world data, are reassuring regarding the pancreatic safety of the GLP-1Ra drug class (32–35).

Moreover, as the frequency of ACH case reports is increasing, one might question whether the chronic use of such therapies over long periods could be involved in the upsurge of the condition (36).

As a matter of fact, pNETs appearing in the context of reactive ACH, both in humans and animals, are slow-growing tumors, often measuring only a few centimeters at the time of diagnosis. These commonly manifest later in life, particularly in middle-aged patients who may have had ACH for many years prior to diagnosis (37, 38). The surge of a glucagon-producing NET in the absence of the typical glucagonoma syndrome triad on an ACH background further supports the hypothesis that ACH could be a precursor of pNET.

## 4 Conclusions

The incidental finding of a clinically non-functioning pNET in a patient with T2D, unexpectedly diagnosed as a glucagon-producing NET on an ACH background, is herein reported. The concomitant presence of T2D and glucagon levels above normal, despite the absence of a typical glucagonoma syndrome triad, were the key for performing glucagon immunostaining in a clinically non-functioning pNET, without which the diagnosis of glucagon-producing NET would easily have been missed. This case highlights the need to consider the diagnosis of glucagon-producing NET on an ACH background, even in the absence of glucagonoma syndrome. Moreover, this case report reinforces the need to further explore the hypothesis of ACH as a potential precursor of glucagon-secreting pNETs or even glucagonomas.

## 5 Patient perspective

The patient took part in the decision process of opting for surgical removal of the 25 mm pNET that, given the small size, could have been kept under surveillance and was pleased to have made that informed choice after understanding the diagnosis.

## Data Availability

The original contributions presented in the study are included in the article. Further inquiries can be directed to the corresponding author.

## References

[1] Kimura W Kuroda A Morioka Y. Clinical pathology of endocrine tumors of the pancreas. Anal Autopsy Cases Dig Dis Sci. (1991) 36:933–42. doi: 10.1007/bf01297144 2070707

[2] Ouyang D Dhall D Yu R. Pathologic pancreatic endocrine cell hyperplasia. World J Gastroenterol. (2011) 17:137–43. doi: 10.3748/wjg.v17.i2.137 PMC3020366 21245985

[3] Yu R. Pancreatic α-cell hyperplasia: facts and myths. J Clin Endocrinol Metab. (2014) 99:748–56. doi: 10.1210/jc.2013-2952 24285676

[4] Henopp T Anlauf M Schmitt A Schlenger R Zalatnai A Couvelard A. Glucagon cell adenomatosis: a newly recognized disease of the endocrine pancreas. J Clin Endocrinol Metab. (2009) 94:213–7. doi: 10.1210/jc.2008-1300 18957496

[5] Otto AI Marschalko M Zalatnai A Toth M Kovacs J Harsing J. Glucagon cell adenomatosis: a new entity associated with necrolytic migratory erythema and glucagonoma syndrome. J Am Acad Dermatol. (2011) 65:458–9. doi: 10.1016/j.jaad.2010.04.010 21763589

[6] Brown K Kristopaitis T Yong S Chejfec G Pickleman J. Cystic glucagonoma: A rare variant of an uncommon neuroendocrine pancreas tumor. J Gastrointest Surg. (1998) 2:533–6. doi: 10.1016/s1091-255x(98)80053-x 10457311

[7] Azemoto N Kumagi T Yokota T Kuroda T Koizumi M Yamanishi H. An unusual case of subclinical diffuse glucagonoma coexisting with two nodules in the pancreas: characteristic features on computed tomography. Clin Res Hepatol Gastroenterol. (2012) 36:e43–7. doi: 10.1016/j.clinre.2011.12.003 22239827

[8] Yu R Nissen NN Dhall D Heaney AP. Nesidioblastosis and hyperplasia of alpha cells, microglucagonoma, and nonfunctioning islet cell tumor of the pancreas: review of the literature. Pancreas. (2008) 36:428–31. doi: 10.1097/MPA.0b013e31815ceb23 18437091

[9] Fujita Y Kozawa J Iwahashi H Yoneda S Uno S Eguchi H. Human pancreatic α- to β-cell area ratio increases after type 2 diabetes onset. J Diabetes Investig. (2018) 9:1270–82. doi: 10.1111/jdi.12841 PMC6215948 29570955

[10] Thompson NW Lloyd RV Nishiyama RH Vinik AI Strodel WE Allo MD. MEN I pancreas: a histological and immunohistochemical study. World J Surg. (1984) 8:561–74. doi: 10.1007/bf01654938 6207668

[11] Anlauf M Schlenger R Perren A Bauersfeld J Koch CA Dralle H. Microadenomatosis of the endocrine pancreas in patients with and without the multiple endocrine neoplasia type 1 syndrome. Am J Surg Pathol. (2006) 30:560–74. doi: 10.1097/01.pas.0000194044.01104.25 16699310

[12] Lubensky IA Pack S Ault D Vortmeyer AO Libutti SK Choyke PL. Multiple neuroendocrine tumors of the pancreas in von Hippel-Lindau disease patients: histopathological and molecular genetic analysis. Am J Pathol. (1998) 153:223–31. doi: 10.1016/s0002-9440(10)65563-0 PMC1852951 9665483

[13] Yu R Dhall D Nissen NN Zhou C Ren SG. Pancreatic neuroendocrine tumors in glucagon receptor-deficient mice. PloS One. (2011) 6:e23397. doi: 10.1371/journal.pone.0023397 21853126 PMC3154424

[14] Yu R Ren SG Mirocha J. Glucagon receptor is required for long-term survival: a natural history study of the Mahvash disease in a murine model. Endocrinol Nutr. (2012) 59:523–30. doi: 10.1016/j.endonu.2012.06.006 22951296

[15] Falconi M Eriksson B Kaltsas G Bartsch DK Capdevila J Caplin M. ENETS consensus guidelines update for the management of patients with functional pancreatic neuroendocrine tumors and non-functional pancreatic neuroendocrine tumors. Neuroendocrinology. (2016) 103:153–71. doi: 10.1159/000443171 PMC4849884 26742109

[16] Unger RH Cherrington AD. Glucagonocentric restructuring of diabetes: a pathophysiologic and therapeutic makeover. J Clin Invest. (2012) 122:4–12. doi: 10.1172/jci60016 22214853 PMC3248306

[17] Lee YH Wang MY Yu XX Unger RH. Glucagon is the key factor in the development of diabetes. Diabetologia. (2016) 59:1372–5. doi: 10.1007/s00125-016-3965-9 27115412

[18] Jia Y Liu Y Feng L Sun S Sun G. Role of glucagon and its receptor in the pathogenesis of diabetes. Front Endocrinol (Lausanne). (2022) 13:928016. doi: 10.3389/fendo.2022.928016 35784565 PMC9243425

[19] Huypens P Ling Z Pipeleers D Schuit F. Glucagon receptors on human islet cells contribute to glucose competence of insulin release. Diabetologia. (2000) 43:1012–9. doi: 10.1007/s001250051484 10990079

[20] Alexandraki KI Kaltsas GA Grozinsky-Glasberg S. Emerging therapies for advanced insulinomas and glucagonomas. Endocr Relat Cancer 30(9). (2023). doi: 10.1530/erc-23-0020 37343152

[21] Hofland J Kaltsas G de Herder WW. Advances in the diagnosis and management of well-differentiated neuroendocrine neoplasms. Endocr Rev. (2020) 41:371–403. doi: 10.1210/endrev/bnz004 31555796 PMC7080342

[22] Soga J Yakuwa Y. Glucagonomas/diabetico-dermatogenic syndrome (DDS): a statistical evaluation of 407 reported cases. J Hepatobiliary Pancreat Surg. (1998) 5:312–9. doi: 10.1007/s005340050052 9880781

[23] Chastain MA. The glucagonoma syndrome: A review of its features and discussion of new perspectives. Am J Med Sci. (2001) 321:306–20. doi: 10.1097/00000441-200105000-00003 11370794

[24] Nauck MA Quast DR Wefers J Pfeiffer AFH. The evolving story of incretins (GIP and GLP-1) in metabolic and cardiovascular disease: A pathophysiological update. Diabetes Obes Metab. (2021) 23 Suppl 3:5–29. doi: 10.1111/dom.14496 34310013

[25] Brubaker PL Drucker DJ. Minireview: Glucagon-like peptides regulate cell proliferation and apoptosis in the pancreas, gut, and central nervous system. Endocrinology. (2004) 145:2653–9. doi: 10.1210/en.2004-0015 15044356

[26] Dankner R Murad H Agay N Olmer L Freedman LS. Glucagon-like peptide-1 receptor agonists and pancreatic cancer risk in patients with type 2 diabetes. JAMA Network Open. (2024) 7:e2350408–e2350408. doi: 10.1001/jamanetworkopen.2023.50408 38175642 PMC10767614

[27] Cao C Yang S Zhou Z. GLP-1 receptor agonists and pancreatic safety concerns in type 2 diabetic patients: data from cardiovascular outcome trials. Endocrine. (2020) 68:518–25. doi: 10.1007/s12020-020-02223-6 32103407

[28] Vangoitsenhoven R Mathieu C van der Schueren B. GLP1 and cancer: friend or foe? Endocr Relat Cancer. (2012) 19:F77–88. doi: 10.1530/erc-12-0111 22691625

[29] Butler AE Campbell-Thompson M Gurlo T Dawson DW Atkinson M Butler PC. Marked expansion of exocrine and endocrine pancreas with incretin therapy in humans with increased exocrine pancreas dysplasia and the potential for glucagon-producing neuroendocrine tumors. Diabetes. (2013) 62:2595–604. doi: 10.2337/db12-1686 PMC3712065 23524641

[30] Butler PC Elashoff M Elashoff R Gale EA. A critical analysis of the clinical use of incretin-based therapies: Are the GLP-1 therapies safe? Diabetes Care. (2013) 36:2118–25. doi: 10.2337/dc12-2713 PMC3687282 23645885

[31] Cao M Pan C Tian Y Wang L Zhao Z Zhu B. Glucagon-like peptide 1 receptor agonists and the potential risk of pancreatic carcinoma: a pharmacovigilance study using the FDA Adverse Event Reporting System and literature visualization analysis. Int J Clin Pharm. (2023) 45:689–97. doi: 10.1007/s11096-023-01556-2 36977858

[32] Abd El Aziz M Cahyadi O Meier JJ Schmidt WE Nauck MA. Incretin-based glucose-lowering medications and the risk of acute pancreatitis and Malignancies: a meta-analysis based on cardiovascular outcomes trials. Diabetes Obes Metab. (2020) 22:699–704. doi: 10.1111/dom.13924 31750601

[33] Azoulay L Filion KB Platt RW Dahl M Dormuth CR Clemens KK. Incretin based drugs and the risk of pancreatic cancer: international multicentre cohort study. Bmj. (2016) 352:i581. doi: 10.1136/bmj.i581 26888382 PMC4772785

[34] Muhammed A Thomas C Kalaiselvan V Undela K. Risk of pancreatitis and pancreatic carcinoma for anti-diabetic medications: findings from real-world safety data analysis and systematic review and meta-analysis of randomized controlled trials. Expert Opin Drug Saf. (2024) 23:731–42. doi: 10.1080/14740338.2023.2284992 37986140

[35] Ayoub M Faris C Juranovic T Chela H Daglilar E. The use of glucagon-like peptide-1 receptor agonists in patients with type 2 diabetes mellitus does not increase the risk of pancreatic cancer: A U.S.-based cohort study. Cancers. (2024) 16:1625. doi: 10.3390/cancers16091625 38730578 PMC11082986

[36] Yang Z Lv Y Yu M Mei M Xiang L Zhao S. GLP-1 receptor agonist-associated tumor adverse events: A real-world study from 2004 to 2021 based on FAERS. Front Pharmacol. (2022) 13:925377. doi: 10.3389/fphar.2022.925377 36386208 PMC9640975

[37] Wermers RA Fatourechi V Wynne AG Kvols LK Lloyd RV. The glucagonoma syndrome clinical and pathologic features in 21 patients. Medicine. (1996) 75(2):53–63. doi: 10.1097/00005792-199603000-00002 8606627

[38] Kindmark H Sundin A Granberg D Dunder K Skogseid B Janson ET. Endocrine pancreatic tumors with glucagon hypersecretion: a retrospective study of 23 cases during 20 years. Med Oncol. (2007) 24:330–7. doi: 10.1007/s12032-007-0011-2 17873310

